# Different Apples, Same Tree: Visualizing Current Biological and Clinical Insights into CTLA-4 Insufficiency and LRBA and DEF6 Deficiencies

**DOI:** 10.3389/fped.2021.662645

**Published:** 2021-04-28

**Authors:** Laura Gámez-Díaz, Markus G. Seidel

**Affiliations:** ^1^Faculty of Medicine, Center for Chronic Immunodeficiency, Institute for Immunodeficiency, Medical Center, Albert-Ludwigs-University of Freiburg, Freiburg, Germany; ^2^Division of Pediatric Hematology-Oncology, Department of Pediatrics and Adolescent Medicine, Medical University of Graz, Graz, Austria; ^3^Research Unit for Pediatric Hematology and Immunology, Medical University of Graz, Graz, Austria

**Keywords:** primary immunodeficiency (PID), primary immune regulatory disorder (PIRD), inborn error of immunity (IEI), cytotoxic T lymphocyte antigen 4 (CLTA-4), hematopoietic stem cell transplantation (HSCT), lipopolysaccharide-responsive beige-like anchor protein (LRBA), differentially expressed in FDCP6 homolog (DEF6), immune deficiency and dysregulation activity (IDDA) kaleidoscope score

## Abstract

Cytotoxic T lymphocyte antigen-4 (CTLA-4) is a crucial immune checkpoint that is constitutively expressed in regulatory T (Treg) cells. Following T-cell activation, CTLA-4 is rapidly mobilized from its intracellular vesicle pool to the cell surface to control the availability of co-stimulatory B7 molecules, thereby maintaining immune homeostasis. Heterozygous mutations in *CTLA-4* lead to defects in (i) CTLA-4 ligand binding, (ii) homo-dimerization, (iii) B7-transendocytosis, and (iv) CTLA-4 vesicle trafficking, resulting in an inborn error of immunity with predominant autoimmunity. CTLA-4 vesicle trafficking impairment is also observed in patients with lipopolysaccharide-responsive beige-like anchor protein (LRBA) deficiency or the *differentially expressed in FDCP6 homolog* (DEF6) deficiency, caused by biallelic mutations in *LRBA* and *DEF6*, respectively. Therefore, patients with CTLA-4 insufficiency, LRBA deficiency, and—most recently reported—DEF6 deficiency present an overlapping clinical phenotype mainly attributed to a defective suppressive activity of Tregs, as all three diseases reduce overall surface expression of CTLA-4. In this paper, we describe the clinical phenotypes of these immune checkpoint defects, their patho-mechanisms, and visually compare them to other immune regulatory disorders (IPEX syndrome, CD27, and CD70 deficiencies) by using the immune deficiency and dysregulation (IDDA version 2.1) “kaleidoscope” score. This illustrates the variability of the degrees and manifestations of immune deficiency and dysregulation. Patients characteristically present with an increased risk of infections, autoimmune cytopenias, multi-organ autoimmunity, and inflammation, which are often severe and life-threatening. Furthermore, these patients suffer an increased risk of developing malignancies, especially Non-Hodgkin's lymphoma. Successful treatment options include regular administration of soluble CTLA-4-Ig fusion protein, Treg cell-sparing immune suppressants like sirolimus or mycophenolate mofetil, and hematopoietic stem cell transplantation. This mini-review highlights the most relevant biological and clinical features as well as treatment options for CTLA-4 insufficiency and LRBA and DEF6 deficiencies.

## Introduction

Primary immune regulatory disorders (PIRDs) are inborn errors of immunity (IEI) with immune dysregulation [category IV of the international classification of human IEI; (1, 2)], that pose a major challenge to physicians, because they are difficult to diagnose and hard to treat. Although phenotypic features and their constellations may be quite specific for a certain underlying monogenic IEI, patients with PIRDs may manifest at extremely different ages, displaying incomplete clinical penetrance. Often, no genotype-phenotype correlation can be found, requiring completely different management approaches even within one family (3–11). For instance, affected siblings of one patient undergoing hematopoietic stem cell transplantation (HSCT) for a PIRD may be oligosymptomatic, but still require no medical therapy at all. In some other PIRDs, the existence of a targeted therapy allows its continuous administration, enabling complete or good partial remissions to be achieved and maintained for many years, albeit with side effects.

In recent years, tremendous progress has been made to more effectively define, understand, and manage the *insufficiency of cytotoxic T-lymphocyte antigen 4* (CTLA-4), the deficiency of *lipopolysaccharide-responsive beige-like anchor protein* (LRBA), or of *differentially expressed in FDCP6 homolog* (DEF6), which represent typical examples of PIRDs (6, 10, 12–17). The classical autoimmune lymphoproliferative syndromes and diseases with hemophagocytosis and/or EBV susceptibility or with colitis are based on the impairment of various receptors or signal transducers relevant to innumerable, more or less cell-type or tissue-specific immune regulatory elements (2). Other IEIs affect the function of regulatory T (Treg) cells—so-called ‘Tregopathies'—such as *immune dysregulation, polyendocrinopathy, enteropathy, X-linked* (IPEX) syndrome, deficiencies of CD25 or *BTB domain and CNC homolog 2* (BACH2), and gain-of-function mutations in *signal transducer and activator of transcription 3* (*STAT3*) (18). Unlike these other IEIs, it appears as though the lack of functional CTLA-4, LRBA, and DEF6 converge in one common primary molecular mechanism, namely, the reduced surface availability of CTLA-4. The low surface expression of CTLA-4 ultimately results in Treg cell dysfunction and leads to impaired immune homeostasis.

CTLA-4 is an important human negative immune checkpoint, as demonstrated by the therapeutic exploitation of its inhibition, next to PD-1, to augment anti-tumoral immunity [*reviewed in* (19)]. The three entities that negatively affect CTLA-4 function in human disease (CTLA-4 insufficiency and LRBA or DEF6 deficiency) may thus also be summarized under the term *immune checkpoint defects* (20). In this mini-review, we summarize the biological characteristics and highlight clinical implications of these immune checkpoint defects, CTLA-4 insufficiency, and LRBA or DEF6 deficiency.

### Biological Aspects of Impaired CTLA-4 Cell Surface Expression and Immune Homeostasis

#### Three Players, One Control Pathway

**Figure 1:**
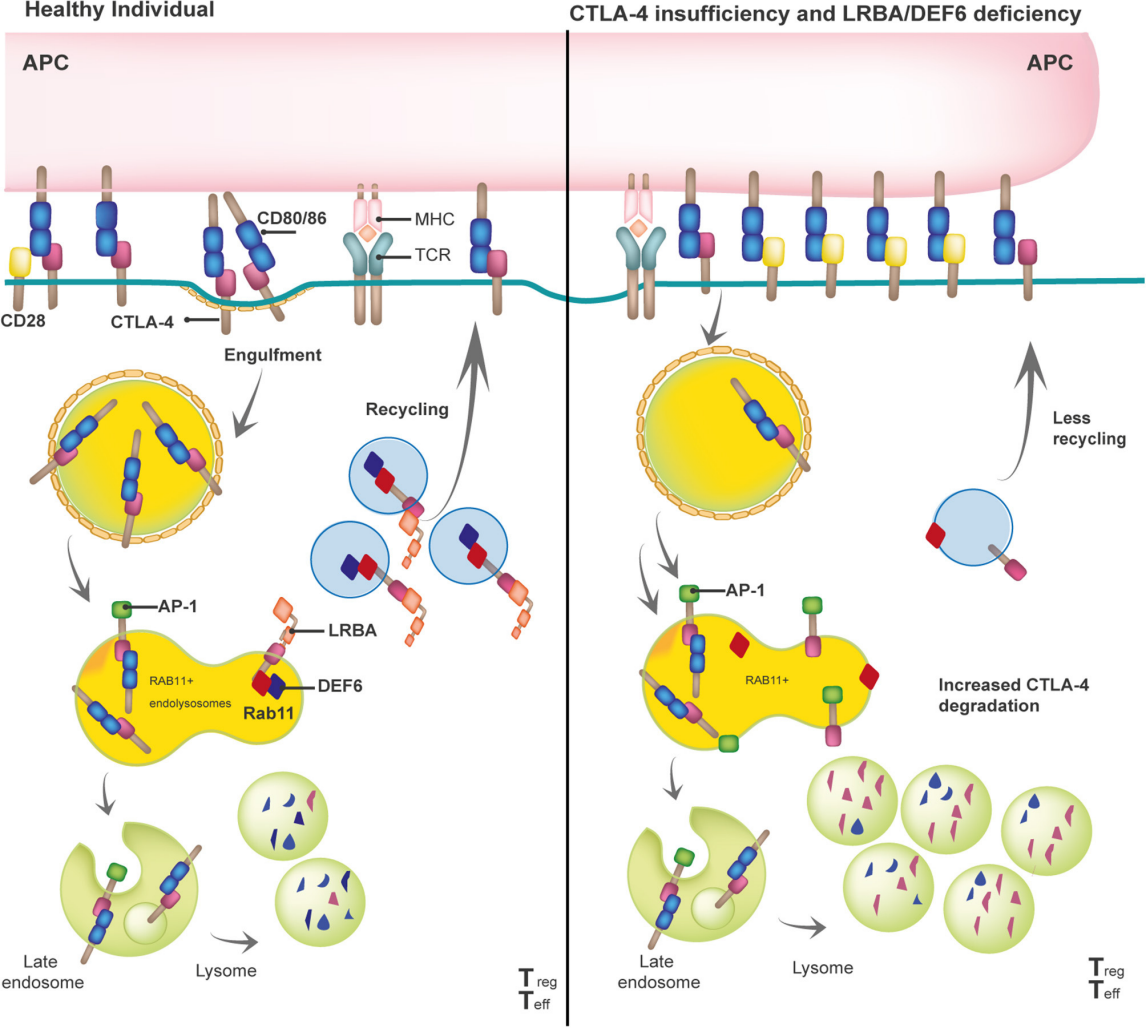
Model of the biological mechanisms involved in the pathophysiology of the three checkpoint defects: CTLA-4 insufficiency, LRBA and DEF6 deficiency.

Given the profound pathophysiological consequences of insufficient or excessive T cell activation, it is not surprising that the adaptive immune response is tightly controlled by positive and negative regulators. Once the T cell receptor (TCR) has engaged with antigenic peptides presented by MHC, CD28, a positive regulator constitutively expressed on the surface of naïve T cells, binds to B7 molecules (CD80 and CD86) that are expressed on antigen-presenting cells (APCs). The CD28/B7 interaction is a vital interaction for immune synapse (IS) formation, as it is required to guarantee full T-cell activation (21). The CD28 homologue protein known as CTLA-4 can “switch off” the T-cell-dependent response after pathogen clearance. Unlike CD28, CTLA-4 is mostly intracellularly stored in clathrin-coated vesicles (CCV) in naïve T cells or constitutively expressed on the surface of Tregs. Upon TCR-dependent activation, CTLA-4-containing CCVs are rapidly mobilized to the cell surface to form homodimers that outcompete CD28 and bind to B7 molecules with higher affinity and avidity. This binding activates cell-intrinsic and cell-extrinsic inhibitory mechanisms that depend on CTLA-4 (22, 23). One of the latter mechanisms (i.e., transendocytosis) physically removes B7 molecules from APCs then effects the internalization and a coordinated destination for either (i) the lysosomal degradation of CTLA-4/B7 complexes or (ii) CTLA-4 receptor recycling (Figure 1). This process depletes the amount of co-stimulatory signals present on the APC's surface, controlling T-cell activation. While the interaction between CTLA-4 and the adaptor protein complex 2 (AP-2) directs the lysosomal degradation of the bound B7 ligands, DEF6 and LRBA essentially control the recycling fate of the CTLA-4 CCVs (15, 16). Upon T-cell activation, mechanistic effects cause LRBA to be intracellularly expressed in Rab11^+^ vesicles, where it binds to the YVKM motif of the cytoplasmic tail of CTLA-4–the same binding site of AP-2. One interesting aspect of this process is that DEF6, a guanine nucleotide exchange factor (GEF), also localizes at the CTLA-4 CCVs while transforming Rab11 to its GTP-bound active state (24). Active Rab11 facilitates the recruitment of many effector proteins that are involved in several membrane trafficking events, such as vesicle budding, transport, tethering, docking, and fusion along the recycling route (25). Researchers still need to clarify whether LRBA is one of the effector proteins recruited by Rab11 upon activation *via* DEF6. Thus, DEF6 and LRBA need to jointly function for CTLA-4 to be successfully recycled to the T-cell surface. Of note, LRBA and DEF6 have been associated with other cellular functions that enable a proper immune response, such as autophagy and NFAT activation, respectively (12, 26).

#### The Duality of Immune Dysregulation and Immune Deficiency

The reduction of available CTLA-4 on the T-cell surface is the common end point of (i) aberrant CTLA-4 protein (i.e., due to truncated CTLA-4 protein, defects in the ligand-binding domain, or homodimerization) caused by heterozygous mutations in *CTLA-4* or (ii) enhanced CTLA-4 lysosomal degradation generated by biallelic mutations in *LRBA* or *DEF6* (Figure 1). Although some missense mutations in *CTLA-4* do not affect the CTLA-4 protein expression, most mutations in *CTLA-4, LRBA*, and *DEF6* reduce CTLA-4 protein levels by 50, 50–75, and 50%, respectively (9, 16, 27, 28). In addition, regardless of the mutations in *CTLA-4, LRBA*, and *DEF6*, Tregs and activated conventional T (Tcon) cells present with an overall but variable reduction in CTLA-4-dependent transendocytosis. Therefore, researchers should consider conducting an *in vitro* evaluation of transendocytosis in T cells from patients suspected of having defects in the CTLA-4 pathway, then evaluate the LRBA and DEF6 protein levels to obtain a differential diagnosis (6, 16, 17). Further correlations observed between the levels of CTLA-4 and the percentage of CTLA-4 transendocytosis or with the clinical onset or clinical severity in these three immune checkpoint deficiencies should be addressed.

Aberrant cell-extrinsic mechanisms (such as lowering IL-2 production, preventing lipid raft formation for TCR signaling, T-cell anergy induction *via* PI3K), and cell-intrinsic mechanisms that are dependent of CTLA-4 lead to a general dysfunction in the suppressive capacity of Tregs. For example, this dysfunction is observed in patients with mutations in *FOXP3*. (These patients suffer from IPEX- Immunodysregulation polyendocrinopathy enteropathy X-linked syndrome) (29). The abnormal CTLA-4 functionality results in (i) immune homeostasis disruption as evidenced by prolonged T-cell activation and migration, causing multiorgan lymphocytic infiltration and (ii) a breakdown of the peripheral immune tolerance as illustrated by the higher circulation of autoreactive lymphocytes, accounting for the autoimmunity development. Increased effector T-cell counts, lymphocytic organ infiltration, as well as multiple types of autoimmunity are commonly observed in patients with CTLA-4 insufficiency, LRBA deficiency, or DEF6 deficiency (6, 9, 16, 30). Autoimmune manifestations are frequently the first symptom in LRBA- and DEF6-deficient patients. Although downstream events of TCR and CD28 signaling assure immunoglobulin production, the loss of CTLA-4 and, therefore, the abundance of CD28, causes the opposite effect. Hence, patients with CTLA-4 insufficiency or LRBA/DEF6 deficiency frequently present with low B-cell counts and hypogammaglobulinemia. Researchers have suggested that these humoral abnormalities occur as a consequence of gradual B cell exhaustion due to over- and/or chronic stimulation (as evidenced by the expansion of CD21^low^ B cells) or due to bone marrow niche disruption following T-cell infiltration (13, 14). Notably, patients with CTLA-4 defects present with CD21^low^ B-cell enrichment in autoreactive clones, as well as increased cTFH cells (31–33). This indicates that CTLA-4 plays an additional potential role in maintaining B-cell homeostasis and preventing the development of autoantibodies (32, 34), just as Tregs control the antigen-specific expansion of Tfh *via* CTLA-4 which, in turn, controls the B-cell response (32, 35, 36).

While patients with germline mutations in *CTLA-4, LRBA* and *DEF6* commonly display features such as immune dysregulation and immune deficiency, mice with similar genetic defects are relatively healthy, even upon infectious challenging or aging. In particular*, Ctla4*-null mice develop fatal autoimmunity early on in life, whereas their *Ctla4*^+/−^ littermates do not develop features of autoimmunity (37). Similarly, *Lrba*-null mice do not present signs of immune dysregulation or aberrant humoral response, despite their loss of LRBA protein and up to 60% reduction in CTLA-4 protein (38). Interestingly, *Lrba*-null mice displayed reduced B-1a cells along with decreased IL-10 production. Recent studies have shown that loss of CTLA-4 expression in B-1a cells results in spontaneous development of autoantibodies, Tfh cells and germinal center in spleens leading to an autoimmune pathology later in life. Whether the expression of CTLA-4 by B-1a cells is also important for immune tolerance in humans is still unknown (39).

Moreover, in 2019, one group reported the development of DSS-induced colitis in *Lrba*-null mice as a consequence of a hyperactivation of the endosomal TLR signaling (40). Whether these discrepancies depend on the mouse background or a specific antigen trigger is still a topic of research. The development of autoimmunity in *Def6*^−/−^ mice does not offer any clues, as some groups reported enhanced susceptibility to the development of an early onset rheumatoid-arthritis-like joint disease or a spontaneous systemic autoimmune disorder (41, 42) whereas others reported inflammation resistance in the *Def6*^−/−^ mouse models (26). Thus, the different phenotypes observed in human and mice in the context of these three PIRDs, despite the fact that CTLA-4, LRBA and DEF6 proteins have the same biological functions in both species, indicate that mice may rely less on the CTLA-4 route to maintain immune homeostasis, or that the murine immune system is more resistant to developing immune dysregulation. Hence, murine data shall be taken cautiously when comparing it to patients with any of these three immune disorders.

#### Malignancies and Immune Checkpoint Inhibition by “Artificial or Natural” Means

One of the mechanisms that tumor cells have adopted to evade immune responses takes advantage of the negative immune checkpoints—like CTLA-4—to induce T-cell anergy or dysfunction. Therefore, novel cancer therapies based on the inhibition of the negative immune checkpoints using receptor-blocking antibodies that enhance antitumor responses have shown durable clinical responses and exceptional therapeutic benefits in multiple types of malignancies (43). In particular, ipilimumab, a monoclonal antibody used against CTLA4, has demonstrated a significant increase in the long-term survival of patients with advanced melanoma (44, 45). Paradoxically, patients who display a “natural” inhibition of CTLA-4, and especially CTLA-4-insufficient patients and DEF6-deficient patients, have an increased risk for malignancies, frequently EBV-associated lymphomas and gastric cancers (17, 46). Lymphopenia, chronic inflammatory tissue, or a pathogen-associated trigger (as for EBV), could account for the development of malignancy in these patients. EBV viral loads were found to be high in patients with mutations in *CTLA-4* and *DEF6*, suggesting either the presence of a susceptibility to EBV reactivation or the development of the onset due to a trigger like EBV. On the other hand, a recently and interesting call for a reappraisal of the CTLA-4 checkpoint blockade was made based on experimental findings that show that anti-CTLA-4 antibody ipilimumab blocks neither B7 transendocytosis by CTLA-4 nor CTLA-4 binding to immobilized or cell –associated B7 (47). Instead, the authors of this study suggested that ipilimumab causes local Treg depletion within the tumor environment through antibody-dependent cellular cytotoxicity (ADCC) and highlighted the critical role of FCR (47). Notably, two-thirds of cancer patients treated with ipilimumab experience side effects that are characteristic symptoms experienced by CTLA-4-insufficient patients, including colitis (48, 49).

It is interesting to note that the upregulation of LRBA and DEF6 mRNA has been observed in different tumor types. In particular, increased levels of DEF6 protein correlate with a poor prognosis in patients with renal cell carcinoma (50). These results could be explained biologically by the fact that DEF6 is also involved in TCR signaling, and specifically in the activation of NFAT through Ca^2+^ release. These observations also might indicate a role for LRBA in the suppression of apoptosis, thereby facilitating cell proliferation and cell survival (12, 51). Finally, CTLA-4 may be expressed by infiltrating Tregs or exhausted Tcon in tumor lesions. Although few studies have described the prognostic value of CTLA-4 levels in the tumor site, its expression has been associated with decreased survival in patients with nasopharyngeal carcinoma and increased survival in those with non-small-cell lung cancer (52).

### Clinical Manifestations and Treatment Options

**Figure 2:**
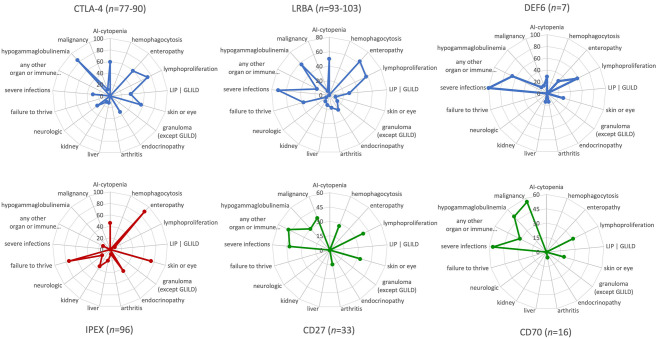
Phenotypic characteristics and differences between CTLA-4 insufficiency, LRBA and DEF6 deficiencies, and other “classic” primary immune regulatory disorders as visualized by the immune deficiency and dysregulation activity (IDDA) “kaleidoscope” score. The phenotypic characteristics of the diseases indicated above in the spider web plots were taken from published large cohort reviews (CTLA-4, LRBA deficiency, CD27 and CD70 deficiency) (6, 9, 10) or small case series of seven patients with DEF6 deficiency (16, 17) and compared to immune dysregulation, polyendocrinopathy, enteropathy, X-linked (IPEX syndrome) (8) as Tregopathy and to CD27 and CD70 deficiency (4, 53) as contrasting primary immune regulatory disorders. The patient numbers presented in the title of each plot vary slightly regarding some features that were not available from all patients, but are always presented as a percentage on 17 *y*-axes arranged in a circle. Within the regular (22-parametric) IDDA score originally developed for LRBA deficiency (11), each criterion is semi-quantified per patient from 0–4°. The full-length *y*-axis titles are: *AI-cytopenia; hemophagocytosis | HLH (according to clinical AND lab criteria of the Histiocyte Society); enteropathy | IBD (inflammatory bowel disease); lymphoproliferation | splenomegaly | hepatomegaly; parenchymal lung disease | LIP (lymphocytic interstitial pneumonitis)| GLILD (granulomatous lymphocytic interstitial lung disease); skin or eye manifestations | eczema, uveitis, alopecia, vitiligo, other; granulomatous disease in any organ (other than GLILD); endocrinopathy | IDDM (insulin-dependent diabetes mellitus), thyroiditis, other; arthritis | other musculoskeletal manifestations; AI-hepatitis | cholangitis | pancreatitis; glomerulonephritis | nephropathy, tubulopathy; neurologic manifestations; failure to thrive | malresorption, wasting; severe infections | opportunistic (excl. asymptomatic chronic infestation; excluding “EBV-susceptibility”); any other organ or immune dysfunction/malady* (e.g., *cardiomyopathy, kidney failure, autoinflammation, allergy); hypogammaglobulinemia and/or immunoglobulin substitution therapy; malignancy, lymphoma (separately added to IDDA score, not included in the score calculation)*.

Several clinical features, including the age of onset, degree of immunodeficiency, severity of the immune dysregulation phenotype with autoimmune and (fewer) autoinflammatory manifestations, and the risk of malignancy, have been investigated in relatively large cohorts of patients with CTLA-4 insufficiency and LRBA deficiency; and similar features have only been examined in two very small patient series with the newly discovered DEF6 deficiency most recently (5–7, 9, 10, 16, 17). Here, we summarize the similarities and some of the fine differences between each of these three immune checkpoint defects. In addition, we provide a visual comparison between the phenotypic features of CTLA-4 insufficiency and LRBA/DEF6 deficiency as checkpoint defects on one hand with other IEI with immune dysregulation, such as IPEX syndrome representing a “classical Tregopathy” and, as a contrast, two PIRDs that are linked to EBV susceptibility and malignancies and, as receptor-ligand pair, share the same pathomechanism, namely CD27 and CD70 deficiencies (Figure 2).

#### Phenotype Variance as Expressed by the Clinical Immune Deficiency and Dysregulation Activity (IDDA Version 2.1) “Kaleidoscope” Score

Mild differences in the clinical phenotypes of CTLA-4, LRBA, and DEF6 exist. CTLA-4 insufficiency is an autosomal dominant disease with ~70% clinical penetrance. In contrast, autosomal recessive inheritance and high levels of disease penetrance but variable disease expressivity are present in LRBA and DEF6 deficiencies. Although their main immunological mechanisms appear to converge in terms of the cell surface expression and availability of CTLA-4 for T-cell regulation and Treg cell function, additional features are expected that are dependent on the tissue-specific expression and physiological functions of the three different proteins. For instance, LRBA is required for proper hearing (as it is highly expressed in the cochlear hair cells) and kidney function in a Lrba-KO mice, yet hearing loss or kidney dysfunction are rarely known features of LRBA deficiency, which was even progredient in one case after HSCT (11, 54). Intriguingly, DEF6 deficiency may present with the occurrence of allergies and/or inflammations and cardiological features (2 out of 7 patients described). These might be due to additional aberrant biological events that depend on DEF6 instead of the impaired CTLA-4 homeostasis pathway (16, 17). Using the new version of the IDDA kaleidoscope score—a 22-parameter scale initially developed to assess the disease activity and burden of LRBA deficiency—we re-evaluated the observed phenotypes and semi-quantified their occurrence in CTLA-4 insufficiency and LRBA/DEF6 deficiency. The new IDDA version includes 14 typical manifestations of immune dysregulation (now including hemophagocytosis for a broader utility in PIRDs), seven other physician-reported factors that indicate the quality of life and need for supportive care, and the occurrence of malignancies [*manuscript in preparation:* (55)]. The IDDA score can be used to compare each parameter on a 2–5-digit scale semi-quantitatively within one individual over time (clinical course, longitudinally) or between individuals or cohorts at a certain time point (cross-sectionally). The “kaleidoscope” function of the IDDA score visualizes the frequency of reported features (without quantifying them) per patient cohort, thereby enabling the comparison of main phenotypical characteristics between different disorders. As expected from the shared pathomechanism, the IDDA kaleidoscope score patterns of CTLA-4 insufficiency, LRBA, or DEF6 deficiency are quite similar, but differ markedly from those with IPEX syndrome (Figure 2, lower left) or of the CD27/CD70 deficiencies (Figure 2, lower center and right panels; please see the Figure legend for data sources). The latter two, being a receptor/ligand pair, resemble each other closely (Figure 2, lower center and right panels).

Researchers are still uncertain why individuals with the same mutation—even siblings or parents and children in one family—sometimes have clinical presentations that vary tremendously regarding their timing of onset and severity of organ involvement, while they present at a very similar age with an almost identical pattern of clinical features in other families. We may only speculate that similar to other diseases with immune dysregulation or autoimmunity, an array of additional disease-modifying factors, such as single nucleotide polymorphisms (SNP) in signaling pathways (e.g., cytokine and cytokine receptor genes or second messengers), epigenetic modifiers of immune tolerance (e.g., the epithelial microbiome and its interaction with HLA-haplotypes), or specific infection triggers and their timing in the context of the maturation of the immune system may play roles that cause this diversity (56–59).

#### Treatment Options and Challenges

Along with symptom-directed therapies such as anti-infective antimicrobial treatment, anti-inflammatory treatment, first-line immunosuppressive treatment against autoimmune cytopenias (60), inflammatory parenchymal lung disease (61), or Treg-sparing immunosuppression in general, many patients who suffered from any of the three described checkpoint defects could be successfully treated with mechanistic target of rapamycin (mTOR) inhibitors, soluble CTLA-4-Ig (abatacept, belatacept), or with allogeneic hematopoietic stem cell transplantation (HSCT) (6, 7, 9–11, 16, 62). A retrospective study of 76 patients was carried out to compare the efficiency of different treatment phases in LRBA deficiency and detected significantly reduced disease activity and burden (IDDA scores) under sirolimus, abatacept, or after HSCT (11). In addition to the already existing risk of malignancies like lymphoma or gastric adenocarcinoma detected in patients with checkpoint defects so far, one could anticipate a shift in the risk pattern from these to other malignancies (e.g., carcinoma) in patients receiving long-term immunosuppression, and especially the pharmacological *checkpoint augmentation* CTLA-4-Ig. However, many more patient cases and follow-up years need to be documented to quantify this risk.

A big challenge for the patient families and their physicians is to choose the right time point and setting for HSCT. As it is *per definitionem* a non-severe but often “profound” combined immunodeficiency, no clear-cut, strict, but still a relatively strong indication for HSCT is known, especially in patients with early and severe manifestations, given their high likelihood to depend on life-long immunosuppressive treatment that add to the disease-inherent risk of immune-mediated organ damage, infections, and malignancies (62). In HSCT, those treating the disease should strive to reach a remission-like pre-HSCT disease control state, using a targeted *remission induction therapy* (Tesch and Seidel, EBMT-Annual Meeting 2020, presentation at the Inborn Errors Working Party meeting). *Post*-HSCT, a full donor T-cell chimerism has been shown to be positively linked to the probability of remission, although patient numbers were too small to define a required degree of chimerism (11, 62). These decisions will hopefully be facilitated and guided in near future by studies that compare the natural and pharmacologically influenced course of the disease by using a standardized measure for disease activity (8, 11); ideally within prospective studies like the ongoing, but no longer recruiting, P-CID study (63); the currently ongoing CHAI-morbidity score that is based on 203 CTLA-4 insufficiency patients; the ABACHAI trial that evaluates the safety and efficacy of abatacept in patients with CTLA-4 insufficiency and LRBA deficiency (000972-40 EU Clinical Trial Registry); and various other new modules included in the European Society for Immunodeficiencies (ESID) registry.

## Discussion and Perspectives

Progress in medicine has often depended on making retrospective deductions by comparing non-random observations. In monogenic disorders like inborn errors of immunity, it has been possible to create new definitions for disease entities by carefully documenting clinical observations, including those of anticipated or unexpected therapeutic responses, and combining these observations with molecular findings from current technically possible biological investigations. These definitions are underpinned by an array of newly recognized signaling pathways and pathophysiological mechanisms. Although much knowledge has been gained in the context of the three immune checkpoint defects reviewed herein, we wish to raise three main points of concern. First, loss-of-function mutations in other proteins involved in the complex process of intracellular trafficking and expression of CTLA-4 still need to be identified as causing IEI/PIRDs. Second, mutations in other immune checkpoint molecules (e.g., PD1/PD-L1 or LAG3) will probably give rise to as-yet-undiscovered autoimmunity disorders, unless they impair other, even more vital (immune regulatory) mechanisms such as materno-fetal tolerance. Third, as pointed out in the section on the treatment of immune checkpoint defects, we must consider the fact that targeted pharmacological immunosuppression in these PIRDs may result in checkpoint augmentation, potentially increasing the life-long risk of malignancies. To this end, researchers will be required to carry out prospective observations of the natural disease course, the individual disease burden, standardized scoring of the disease activity and evaluate prognostic and therapeutic biomarkers, a process that is ideally undertaken in the context of international collaborations, to include as many patients as possible (e.g., those listed in the ESID registry and the GAIN study or by performing disease-specific prospective studies) to develop the best management strategies to use with patients with IEI, such as CTLA-4 insufficiency or LRBA or DEF6 deficiencies.

## Author Contributions

LG-D and MS jointly wrote the manuscript and designed the figures.

## Conflict of Interest

The authors declare that the research was conducted in the absence of any commercial or financial relationships that could be construed as a potential conflict of interest. The reviewer SB declared a past co-authorship with one of the authors MS to the handling editor.

## Abbreviations

AP-2: adaptor protein complex
APC: antigen presenting cells
BEACH: Beige and Chediak Highashi
CCV: clathrin-coated vesicles
CTLA-4: cytotoxic T-lymphocyte antigen 4
DEF6: differentially expressed in FDCP6 homolog
EBV: Epstein-Barr-Virus
GEF: guanine exchange factor
IEI: inborn errors of immunity
Ig: immunoglobulin
IPEX: immunodysregulation polyendocrinopathy enteropathy X-linked
IS: immune synapse
LRBA: lipopolysaccharide-responsive beige-like anchor protein
MHC: major histocompatibility complex
PI3K: phosphoinositide 3-kinnase
PIRD: primary immune regulatory disorders
NFAT: nuclear factor of activated T cells
SNP: Single nucleotide polymorphism
TCR: T-cell receptor signaling
TFH: T follicular helper
Tregs: regulatory T cells.
